# Identification of Potential lncRNA-miRNA-mRNA Regulatory Network Contributing to Arrhythmogenic Right Ventricular Cardiomyopathy

**DOI:** 10.3390/jcdd11060168

**Published:** 2024-05-30

**Authors:** Haotong Li, Shen Song, Anteng Shi, Shengshou Hu

**Affiliations:** State Key Laboratory of Cardiovascular Disease, Fuwai Hospital, National Center for Cardiovascular Diseases, Chinese Academy of Medical Sciences and Peking Union Medical College, Beijing 100037, China; lihaotong@fuwai.com (H.L.); songshen@fuwaihospital.org (S.S.); shiatpkuhsc@pku.edu.cn (A.S.)

**Keywords:** arrhythmogenic right ventricular cardiomyopathy, lncRNA-miRNA-mRNA network, ceRNA, diagnostic prediction model

## Abstract

Arrhythmogenic right ventricular cardiomyopathy (ARVC) can lead to sudden cardiac death and life-threatening heart failure. Due to its high fatality rate and limited therapies, the pathogenesis and diagnosis biomarker of ARVC needs to be explored urgently. This study aimed to explore the lncRNA-miRNA-mRNA competitive endogenous RNA (ceRNA) network in ARVC. The mRNA and lncRNA expression datasets obtained from the Gene Expression Omnibus (GEO) database were used to analyze differentially expressed mRNA (DEM) and lncRNA (DElnc) between ARVC and non-failing controls. Differentially expressed miRNAs (DEmiRs) were obtained from the previous profiling work. Using starBase to predict targets of DEmiRs and intersecting with DEM and DElnc, a ceRNA network of lncRNA-miRNA-mRNA was constructed. The DEM and DElnc were validated by real-time quantitative PCR in human heart tissue. Protein–protein interaction network and weighted gene co-expression network analyses were used to identify hub genes. A logistic regression model for ARVC diagnostic prediction was established with the hub genes and their ceRNA pairs in the network. A total of 448 DEMs (282 upregulated and 166 downregulated) were identified, mainly enriched in extracellular matrix and fibrosis-related GO terms and KEGG pathways, such as extracellular matrix organization and collagen fibril organization. Four mRNAs and two lncRNAs, including *COL1A1*, *COL5A1*, *FBN1*, *BGN*, *XIST*, and *LINC00173* identified through the ceRNA network, were validated by real-time quantitative PCR in human heart tissue and used to construct a logistic regression model. Good ARVC diagnostic prediction performance for the model was shown in both the training set and the validation set. The potential lncRNA-miRNA-mRNA regulatory network and logistic regression model established in our study may provide promising diagnostic methods for ARVC.

## 1. Introduction

Arrhythmogenic right ventricular cardiomyopathy (ARVC) (OMIM identifier #609040), discovered in the 1970s, is a hereditary cardiomyopathy that can lead to sudden cardiac death and life-threatening heart failure [1]. It is mainly characterized by local or global enlargement of the right ventricle, a fibrofatty infiltration of the right ventricle which replaces the myocardium and ventricular late potential [2]. Due to the large variability in the clinical manifestations of ARVC, its incomplete penetrance among relatives, and variable gene mutations (more than 25 have been described), the early diagnosis and identification of ARVC is very difficult [1,3,4]. At present, the classical diagnostic methods of ARVC include electrocardiogram, imaging examinations, and pathological diagnosis. Electrocardiograms and imaging examinations can only provide indirect evidence. Pathological examination, as the gold standard, can confirm a diagnosis, but most of the tissues are obtained from end-stage patients [2,3]. Moreover, our group previously proposed the clinical classification of ARVC according to the clinical phenotype and found several biomarkers of ARVC that are more conducive to accurate diagnosis and further treatment [5,6,7]. However, all these methods depend on clinical phenotypes and criteria, which are variable. Therefore, the exploration of the genetic diagnosis method for ARVC is clinically significant.

The competing endogenous RNA (ceRNA), including but not limited to mRNA, long non-coding RNA (lncRNA), and micro-RNA (miRNA), represent a novel gene regulatory model [8]. In brief, miRNA can target mRNA, causing mRNA degradation or inhibiting its translation, and lncRNA can bind miRNA to act as a sponge [9]. Therefore, lncRNA and mRNA competitively bind miRNA to regulate gene expression. This provides a new perspective for researchers to study the transcriptome and helps to explain some biological phenomena in a more comprehensive and in-depth way. miRNA is a non-coding RNA composed of about 22 nucleotides, and lncRNA is a non-coding RNA with a length greater than 200 nucleotides [9]. Increasing evidence suggests that miRNA and lncRNA are both essential in cardiovascular diseases, including ARVC [10,11,12]. To date, there have been five miRNA profiling studies in ARVC-based human samples: two in plasma, two in right ventricular myocardial tissue, and one in pericardial fluid [13,14,15,16,17]. Several circulating miRNAs, including miR-320a, miR-144-3p, miR-145-5p, miR-185-5p, and miR-494, were identified in the two microarray analyses in plasma and are potential diagnostic biomarkers for ARVC [14,15]. Tissue-based miRNA profiling of ARVC identified several miRNAs that are associated with the Wnt/β-catenin pathway and Hippo pathway, including miR-21-5p, miR-135b, miR-122-5p, miR-142-3p, miR-182-5p, miR-183-5p, miR-133a-3p, and miR-133b [13,16]. For lncRNA and mRNA profiling studies in ARVC, for example, a previous study used right ventricular and left ventricular myocardium of non-failing hearts and ARVC hearts, comparing them to explore the key mRNA and lncRNA for ARVC pathogenesis [18]. However, no systemic and detailed analysis of the lncRNA-miRNA-mRNA regulatory network has been conducted to improve the diagnostic method of ARVC.

In this study, we analyzed the differentially expressed mRNAs, lncRNAs, and miRNAs in ARVC samples compared with the non-failing group, trying to elucidate the potential lncRNA-miRNA-mRNA ceRNA regulatory network of ARVC and identify its diagnostic role.

## 2. Materials and Methods

### 2.1. Dataset Filtering

Expression profiles including mRNA, miRNA, and lncRNA were searched in the GEO database using the keyword “ARVC OR AC” first. Only the datasets whose samples were collected from heart tissue were adopted. Due to the scarcity of ARVC samples, only 3 expression profiles (GSE107475, GSE107156, GSE29819) could be used in this study, which included both mRNA and lncRNA expression data. For miRNA expression data, papers about “miRNA and ARVC” were searched in PubMed; 2 related articles whose samples were from heart tissue were obtained [13,16]. Through evaluating the type of high-throughput technology, one recently published paper was adopted for the miRNA dataset [16]. The GSE107475 and GSE107156 datasets consisted of 9 heart samples from ARVC patients and 5 samples from non-failure hearts as controls, respectively, which were detected by the GPL16791 Illumina HiSeq 2500 platform. The GSE29819 datasets consisted of 6 right ventricular samples of ARVC patients and 6 right ventricular samples of non-failure control hearts, which were detected by the GPL570 [HG-U133_Plus_2] Affymetrix Human Genome U133 Plus 2.0 Array platform. Considering that ARVC mainly involves the right ventricle, only cardiac tissue samples obtained from the right ventricle were included in the current study.

### 2.2. Differential Gene Expression Analysis

Differentially expressed mRNA (DEM) and differentially expressed lncRNA (DElnc) were analyzed in GSE107475 and GSE107156 by limma R package. *p*-value < 0.05 and |log2FC| > 1 were considered as the differentially expressed criteria. For mRNA and lncRNA separation, the human genome reference sequence (Homo_sapiens. GRCh38.104. gtf) was obtained from Ensembl genome browser 104 (https://uswest.ensembl.org/index.html (accessed on 1 November 2021)) to extract “gene_id”, “gene_name”, and “gene_biotype”. If the biotype is “protein_coding”, the term is considered to be mRNA. If the biotype is “lncRNA”, the term is considered to be lncRNA. The volcano plots and the heatmaps were used to visualize DEMs and DElncs by ggpubr R package and ComplexHeatmap R package. Differentially expressed miRNAs (DEmiRs) were obtained from the study which used the cut-offs of log2FC > 1 and <−1, respectively, and adjusted *p*-value < 0.05.

### 2.3. GO and KEGG Pathway Enrichment Analyses

Gene ontology (GO) analysis, which includes biological process, cellular component and molecular function, and Kyoto Encyclopedia of Genes and Genomes (KEGG) pathway enrichment analysis were performed by using clusterProfiler R package to determine the biological functions of the DEMs [19]. GO enrichment terms with *p*-value < 0.05 and *q*-value < 0.05 were considered as statistically significant. Cut-offs of KEGG enrichment terms were *p*-value < 0.05 and *q*-value < 0.05.

### 2.4. Protein–Protein Interaction Network Construction

The proteins association network of DEMs was constructed by STRING (http://www.string-db.org (accessed on 15 November 2021)). Cytoscape (v3.7.2) was used to visualize the network. Molecular Complex Detection (MCODE) (version 1.6.1), a plugin of Cytoscape was chosen to identify modules in the network. The modules with MCODE score ≥4.0 and nodes ≥6 were considered to be significant. Genes in all the significant modules were regarded as the hub genes.

### 2.5. Weighted Gene Co-Expression Network Analysis (WGCNA)

To perform WGCNA, 3829 genes in differential expression analysis with *p* < 0.05 were selected. First, the adjacency matrix was calculated for the selected genes, and then converted to a topological overlap matrix to reduce noise and false correlation. The gene modules were identified according to the topological overlap matrix by the dynamic shearing method. After that, the eigenvector value of each module was calculated in turn, then cluster analysis on the modules was performed, and the closer modules were merged into a new module. Key modules were identified by setting the soft thresholding power of 9, cut height of 0.25, and minimal module size of 30. Absolute values of gene significance (GS) > 0.6 and module membership (MM) > 0.9 were considered to define the hub genes in key modules.

### 2.6. lncRNA-miRNA-mRNA Network Construction

DEMs, DElncs, and DEmiRs were used to construct lncRNA-miRNA-mRNA network. First, we obtained miRNA-mRNA and miRNA-lncRNA pairs by predicting DEmiR targets. StarBase (https://rnasysu.com/encori/ (accessed on 15 November 2021)) was used to predict targeted mRNA and lncRNA [20,21]. In starBase, CLIP-Data ≥ 3 and programNum ≥ 2 were selected to obtain refined results with high stringency. Second, the predicted mRNA and lncRNA were intersected with DEMs and DElncs. Considering that the interacting miRNA and mRNA expression are negatively correlated, up-regulated DEMs were intersected with the predicted mRNA of down-regulated DEmiRs, and the down-regulated DEMs were intersected with the predicted mRNA of up-regulated DEmiRs. Finally, DEmiR-DEM and DEmiR-DElnc pairs were put into Cytoscape to construct and visualize the network.

### 2.7. Logistic Regression Model

Logistic regression is a generalized linear regression analysis model that can predict the probability of disease occurrence based on risk factors, so it can be used to construct a diagnostic prediction model. Here, we used the disease group and the healthy group as the binary dependent variable, and the gene expression as the continuous independent variable. Through logistic regression analysis, the weights of key mRNA and lncRNA were obtained, which can be used to predict ARVC disease diagnosis. The glm function in the stats R package and the crPlots function in the car R package were used to construct the logistic regression model. A training set (*n* = 14, 9 ARVC and 5 controls) and a validation set (*n* = 12, 6 ARVC and 6 controls) were used to establish and testify this model and their ROC curves were plotted by pROC R package.

### 2.8. Real-Time Quantitative PCR (qPCR)

For the qPCR assay, total mRNA was extracted from human right ventricular heart tissue of ARVC patients or donors using TRIzol reagent (Invitrogen, Waltham, MA, USA). The informed consent for research use of donor and explanted ARVC hearts was obtained before harvesting donor hearts and heart transplantation. The total mRNA was quantified using a Nanodrop 2000 (Thermo Fisher Scientific, Madison, WI, USA) and 1000 ng RNA was reverse-transcribed into cDNA using PrimeScript RT Master Mix (Takara, RR036A, Tokyo, Japan). SYBR Green PCR Master Mix (Applied Biosystems, Waltham, MA, USA) was used to quantify the PCR products by Vii7 Real-Time PCR System (Applied Biosystems, Waltham, MA, USA). The primers used in the experiment are provided in Supplementary Table S2.

### 2.9. Dual-Luciferase Reporter Assay

Dual-luciferase reporter was obtained using a Dual-luciferase reporter assay system kit (Promega, Madison, WI, USA). Briefly, AC16 cells (a human ventricular cardiomyocyte cell line) were co-transfected with human key gene promoter reporter plasmid (pGL3-NDRG2-Luc) plus the Renilla luciferase reporter plasmid (pRL-TK) with Lipofectamine 2000 (Invitrogen, Camarillo, CA, USA) following the manufacturer’s instructions. The reporter luciferase activities were determined as the results of reporter activities divided by Renilla activities and normalized. All the transfection experiments were performed in triplicate and repeated at least three times independently.

### 2.10. Statistics

All values are presented as mean ± SEM. Two-group comparisons were performed with an unpaired, 2-tailed Student’s *t*-test. A value of *p* < 0.05 was considered statistically significant.

## 3. Results

### 3.1. Differentially Expressed mRNAs, lncRNAs, and miRNAs in ARVC

**Figure 1:**
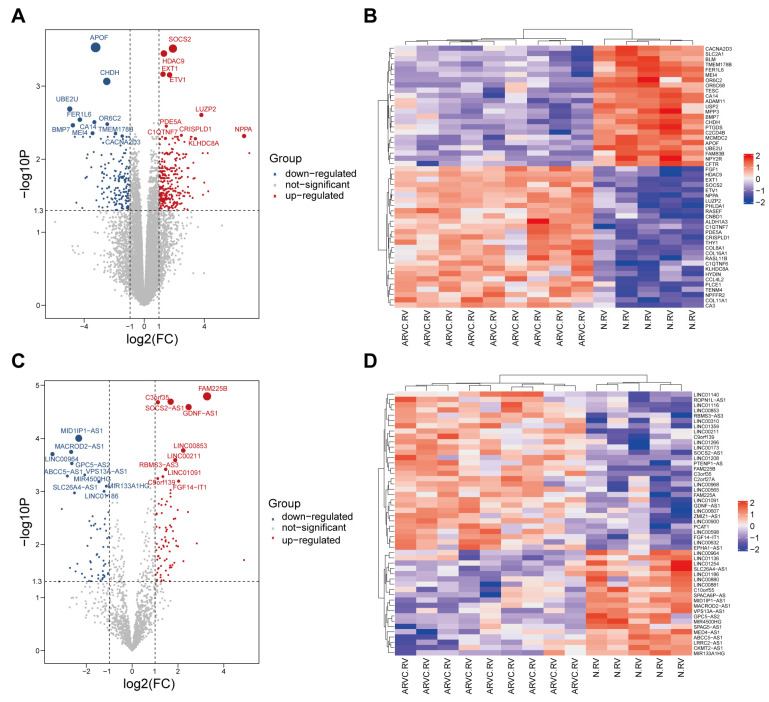
Differentially expressed mRNAs (DEMs) and lncRNAs (DElnc) between ARVC and non-failing control. (**A**,**B**) Volcano plot of DEMs (**A**) and heatmap of top 50 DEMs (**B**) from GSE107475 and GSE107156. (166 genes down-regulated, 282 genes up-regulated). (**C**,**D**) Volcano plot of DElncs (**C**) and heatmap of top 50 DElncs (**D**) from GSE107475 and GSE107156. (60 lncRNAs down-regulated, 79 lncRNAs up-regulated). FC, Fold change.

Through comprehensive search and screening, a total of 27 ARVCs and 17 healthy controls were included in this study (Table 1). GSE107475 and GSE107156 were combined and separated into an mRNA and an lncRNA dataset. Then we carried out normalization to reduce technical errors between samples from the mRNA expression dataset (Supplementary Figure S1A,B) and the lncRNA expression dataset (Supplementary Figure S1D,E). After normalization, principal component analysis was performed; the two groups of samples have a good degree of discrimination in both the mRNA dataset (Supplementary Figure S1C) and the lncRNA dataset (Supplementary Figure S1F). By differential gene expression analysis, 448 DEMs between ARVC samples and the control samples, including 282 up-regulated genes and 166 down-regulated genes, were identified from the mRNA dataset (Figure 1A,B). A total of 139 DElncs between ARVC samples and the control samples, including 79 up-regulated lncRNAs and 60 down-regulated lncRNAs, were identified in the lncRNA dataset (Figure 1C,D). Twenty-one DEmiRs between ARVC samples and the control samples, comprising 8 up-regulated miRNAs and 13 down-regulated miRNAs were obtained from the previous study [16] (Supplementary Table S1).

### 3.2. Functional Enrichment Analysis of the DEMs

**Figure 2:**
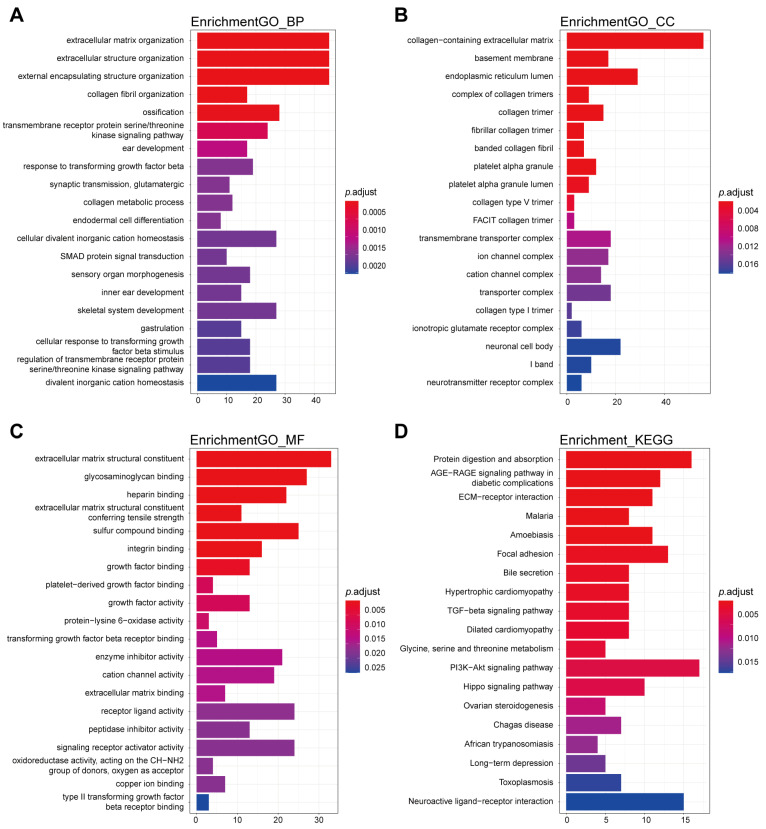
Functional enrichment analysis of DEMs. Gene Ontology (GO) (**A**–**C**), including biological process (**A**), cellular component (**B**), molecular function (**C**), and Kyoto Encyclopedia of Genes and Genomes (KEGG) (**D**), enrichment analysis of all the DEMs. The top 20 significant terms are shown.

To explore the functional enrichment of the 448 DEMs, GO enrichment analysis and KEGG pathway enrichment analysis were performed. The top 20 GO terms including biological process, cellular component, molecular function, and KEGG pathways are shown in Figure 2. Biological process GO terms are most enriched in extracellular matrix and fibrosis-related terms such as extracellular matrix organization and collagen fibril organization (Figure 2A). Meanwhile, the collagen-containing extracellular matrix ranks first as the most significant in cellular component GO terms (Figure 2B), and the extracellular matrix structural constituent ranks first in molecular function GO terms (Figure 2C). According to KEGG analysis, DEMs are most related to protein digestion and absorption, AGE−RAGE signaling pathway in diabetic complications and ECM−receptor interaction (Figure 2D).

**Figure 3:**
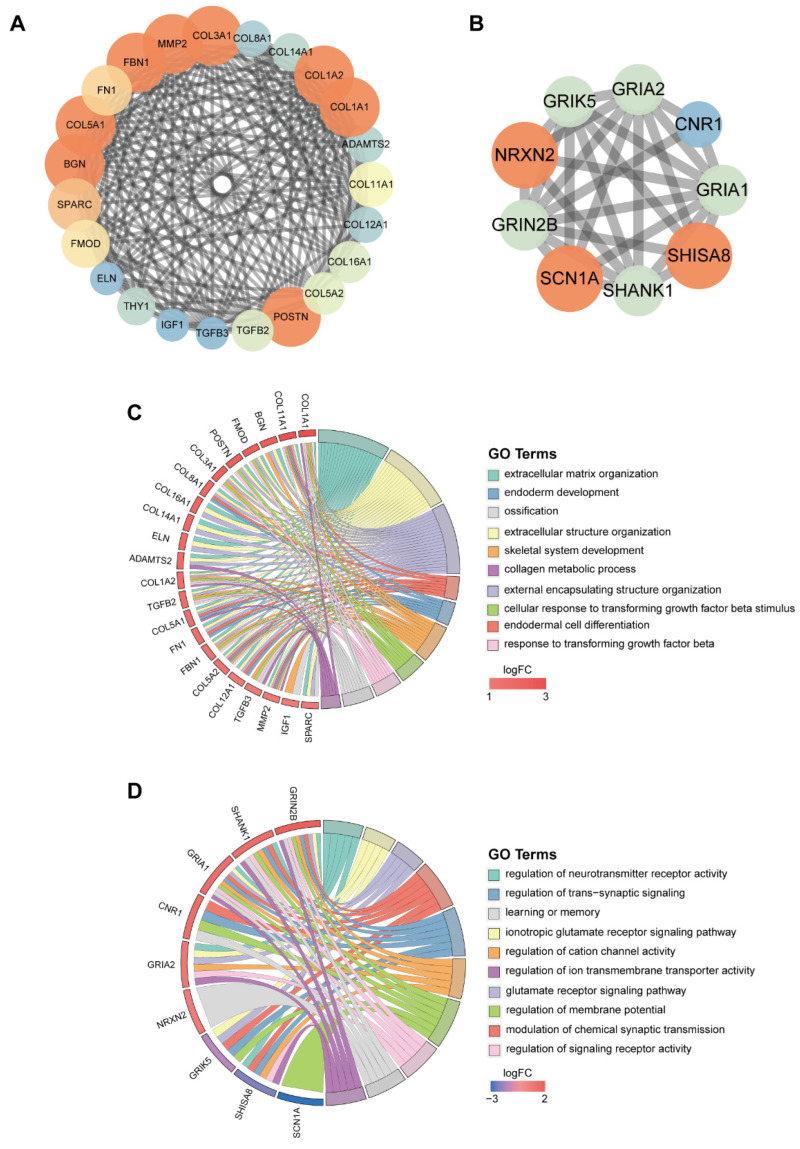
The protein–protein interaction (PPI) network of the DEMs. (**A**,**B**) Top modules identified through MCODE in Cytoscape software (v3.7.2). The size of the circle is positively correlated with the MCODE score and red represent the highest MCODE score while blue represent the lowest MCODE score. (**C**,**D**) GO enrichment analysis of the hub genes of the PPI network in (**A**) (**C**) and (**B**) (**D**). logFC, log2 fold change.

For a better understanding of the most important biological function of the DEMs, the PPI network was constructed, and two key modules were identified (Figure 3A,B). The genes in the one module include *COL3A1*, *MMP2*, *FBN1*, *FN1*, *COL5A1*, *BGN*, *SPARC*, *FMOD*, *ELN*, *THY1*, *IGF1*, *TGFB3*, *TGFB2*, *POSTN*, *COL5A2*, *COL16A1*, *COL12A1*, *COL11A1*, *ADAMTS2*, *COL1A1*, *COL1A2*, *COL14A1*, *COL8A1*, which are associated with the extracellular matrix organization and collagen metabolic process (Figure 3C); the genes in the other module include *NRXN2*, *GRIN2B*, *SCN1A*, *SHANK1*, *SHISA8*, *GRIA1*, *CNR1*, *GRIA2*, *GRIK5*, which are related with the ionotropic glutamate receptor signaling pathway and regulation of cation channel activity (Figure 3D).

### 3.3. Weighted Gene Co-Expression Network Analysis

**Figure 4:**
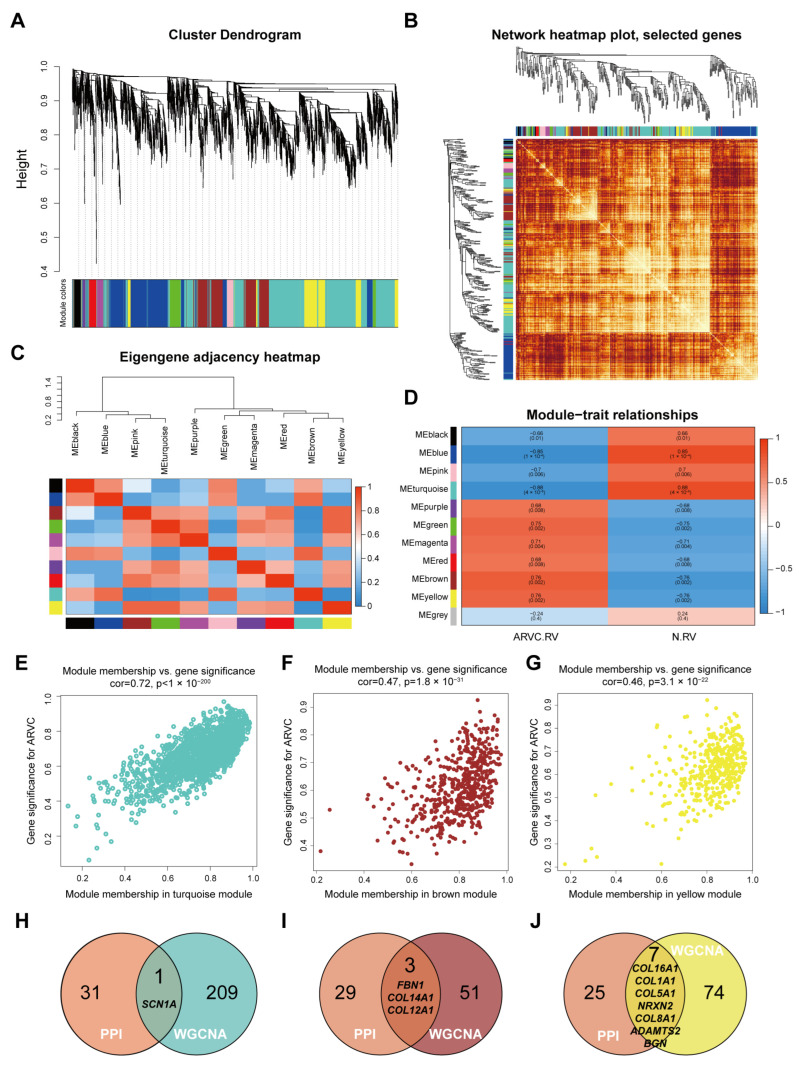
Identification of crucial modules and hub genes associated with ARVC by WGCNA. (**A**) Dendrogram of the DEMs clustered according to the topological overlap matrix. (**B**) Network heatmap of randomly selected genes in each module. (**C**) Eigengene adjacency heatmap of the 10 modules. (**D**) Heatmap showing the relationship between module eigengenes and clinical conditions. The correlation coefficient and *p*-value are shown in the grid. Red means a positive correlation, and blue indicates a negative correlation. (**E**–**G**) Correlation plots of module membership and gene significance of genes in the turquoise (**E**), brown (**F**), and yellow module (**G**). (**H**–**J**) Selected genes in WGCNA modules, including turquoise (**H**), brown (**I**), yellow (**J**) module, were intersected with selected genes in PPI network to identified hub genes, shown in Venn plots.

To further identify hub genes most associated with ARVC, we performed WGCNA between ARVC and control samples. We selected 3829 genes with *p* < 0.05 from the differential expression analysis to cover enough genes for WGCNA. According to the principle of a scale-free network, the weighted value of the correlation coefficient was calculated and the best beta value was 9 (Supplementary Figure S2A,B). A total of 11 modules were identified, including 10 meaningful modules and one grey module with non-clustering DEMs (Figure 4A). Correlations and adjacency relations of modules were calculated, which show good discrimination of the modules by WGCNA (Figure 4B,C). To identify the most important module, we calculated the correlation between the modules and the disease phenotypes. The brown module and the yellow module are the most positively correlated modules with ARVC, meanwhile, the turquoise module is the most negative one (Figure 4D). There are 1550, 548, and 398 genes in the turquoise, brown, and yellow modules, respectively. The top genes of the three modules were selected according to the gene significance and module membership value (Figure 4E–G). Then we took the intersection of the hub gene obtained by PPI and WGCNA; finally, 11 hub genes including *SCN1A*, *FBN1*, *COL14A1*, *COL12A1*, *COL16A1*, *COL1A1*, *COL5A1*, *NRXN2*, *COL8A1*, *ADAMTS2,* and *BGN* were identified (Figure 4H–J).

### 3.4. The Potential lncRNA-miRNA-mRNA Regulatory Network

**Figure 5:**
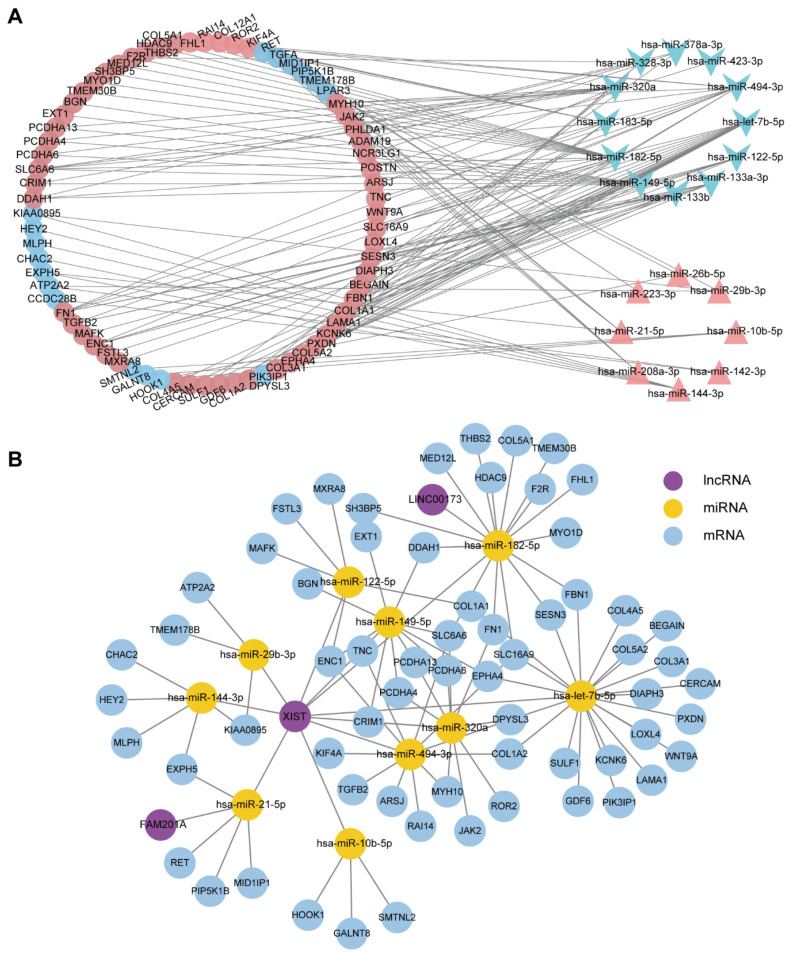
The potential lncRNA-miRNA-mRNA network in ARVC. (**A**) The potential miRNA-mRNA regulatory network in ARVC. The triangles represent up-regulated miRNAs, and the arrows represent down-regulated miRNAs. The circle represents mRNA, in which red represents up-regulation and blue represents down-regulation. (**B**) The potential ceRNA network of lncRNA-miRNA-mRNA in ARVC.

Previous studies showed miRNAs play important roles in ARVC disease progression and meanwhile the ceRNA mechanism is crucial in various cardiovascular diseases. Therefore, we used starBase and lncRNABase to predict the downstream target mRNAs and lncRNAs of DEmiRs, then constructed an lncRNA-miRNA-mRNA ceRNA network of the intersection between predicted targets and differentially expressed genes. First, an miRNA-mRNA network, including 12 down-regulated miRNAs targeting 17 up-regulated mRNAs and eight up-regulated miRNAs targeting 55 down-regulated mRNAs was established (Figure 5A). Then, miRNA and lncRNA pairs were added and the ceRNA network of lncRNA-miRNA-mRNA was obtained (Figure 5B).

**Figure 6:**
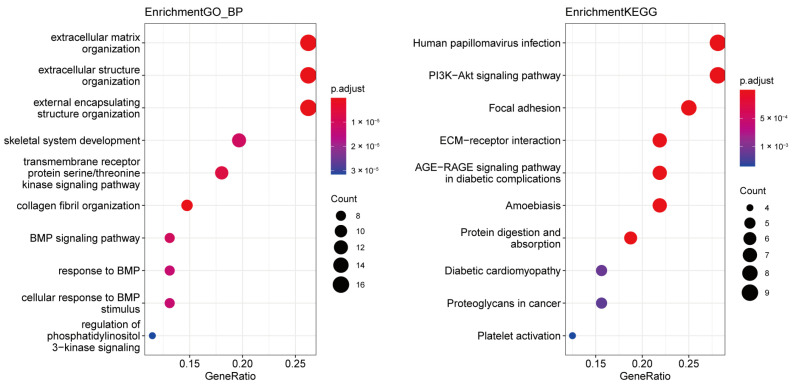
GO and KEGG enrichment analysis of DEMs in the network.

There are three lncRNAs in the network that are *XIST*, *LINC00173,* and *FAM201A*. Notably, *XIST* is the most important lncRNA of the three, which regulates all the 10 miRNAs in the network. However, the other two lncRNAs only interact with one miRNA. To further understand the biological significance of the ceRNA network, we performed GO and KEGG enrichment analyses of the differentially expressed genes in the ceRNA network, which mainly enriched in the extracellular matrix organization and PI3K−Akt signaling pathway (Figure 6). Considering both the ceRNA network and the hub genes analyzed by PPI and WGCNA, four mRNAs (*FBN1*, *COL1A1*, *COL5A1*, *BGN*) and two lncRNAs (*XIST*, *LINC00173*) were identified, and we speculated that the six nodes were potential biomarkers associated with ARVC.

### 3.5. Logistic Regression Model for Prediction of ARVC

**Figure 7:**
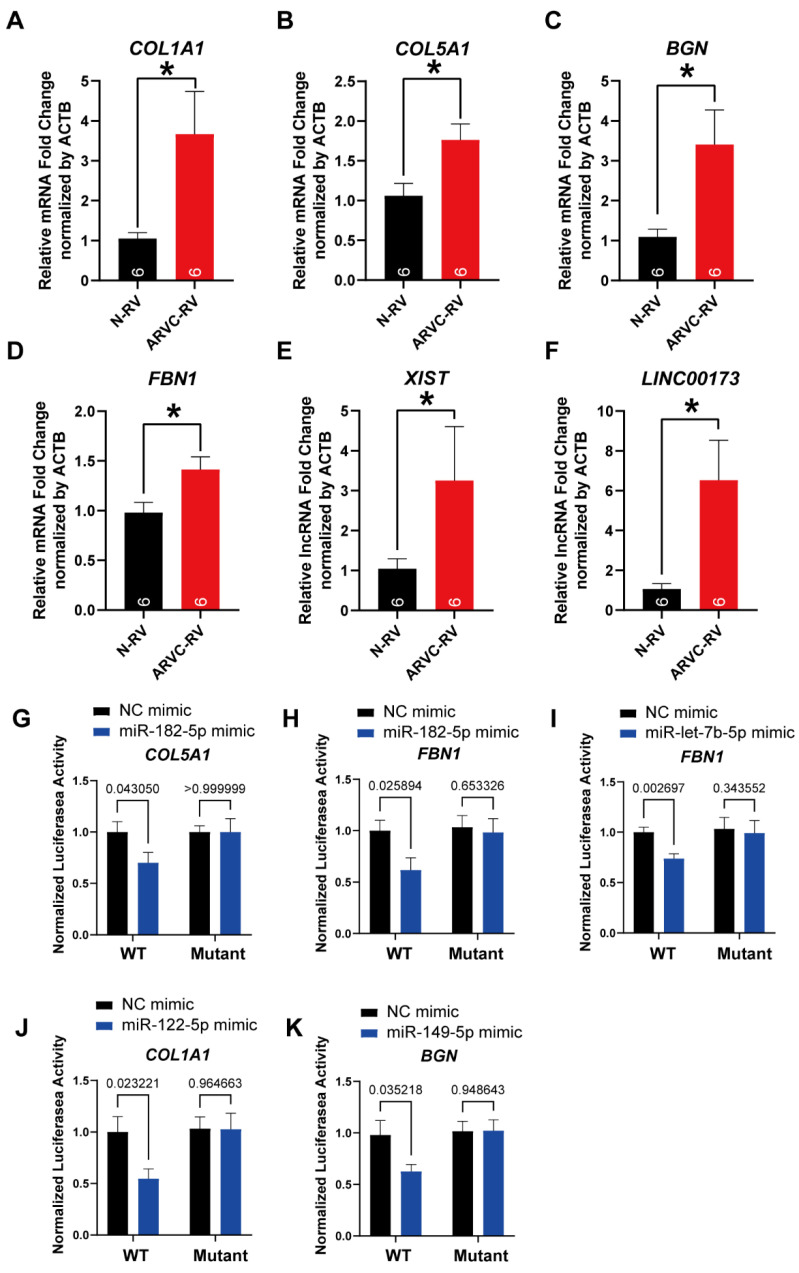
Verification of key gene expression and miRNA-mRNA regulation. (**A**–**F**) Hub mRNA and lncRNA are validated by real-time quantitative PCR in the right ventricular (RV) of human ARVC (ARVC-RV) and non-failing donor heart (N-RV). (**A**–**D**) mRNAs including *COL1A1*, *COL5A1*, *BGN,* and *FBN1* identified from the ceRNA network are up-regulated in ARVC-RV; relative mRNA fold changes are normalized by *ACTB* (*n* = 6 per group). (**E**,**F**) lncRNAs, including *XIST* and *LINC00173* identified from the ceRNA network, are up-regulated in ARVC-RV; relative lncRNA fold changes are normalized by *ACTB* (*n* = 6 per group). (**G**–**K**) Dual-luciferase reporter assay in AC16 cells to validate miRNA-mRNA regulation identified by bioinformatics (*n* = 3 per group). Data are presented as the mean ±SEM, 2-tailed Student *t*-test used, * means *p* < 0.05.

**Figure 8:**
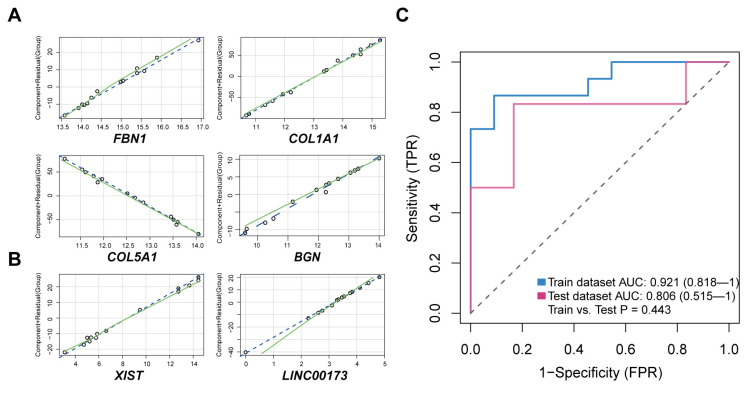
Logistic regression model for ARVC diagnostic prediction. (**A**,**B**) Component plus residual plot of four mRNA (*FBN1*, *COL1A1*, *COL5A1*, *BGN*) (**A**) and two lncRNA (*XIST*, *LINC00173*) (**B**) in the established model. Linear correlation between independent variables and dependent variables indicated that it is suitable to adopt logistic regression. (**C**) The ROC curve of the diagnostic prediction performance of the established model in the training dataset and the test dataset. AUC, the area under the curve.

In order to confirm the diagnostic role of the above biomarkers in ARVC, we constructed logistic models in GSE107475, GSE107156, and GSE29819 datasets based on these four mRNAs (*FBN1*, *COL1A1*, *COL5A1*, *BGN*) and two lncRNAs (*XIST*, *LINC00173*). First, these RNA identified through the ceRNA network were validated by real-time quantitative PCR in human heart tissue; all of them are up-regulated in ARVC-RV (Figure 7A–F). And miRNA-mRNA regulation identified by bioinformatics was validated by dual luciferase reporter assay in AC16 cells (Figure 7G–K). The component residual plot confirms the linear correlation between the dependent variables and independent variables, indicating that the logistic regression model is suitable for the datasets (Figure 8A,B). Due to the sample size not being large enough to apply five-fold cross-validation, we utilized one training set and one validation set to assess the reliability of the established model. The AUC value of the module in the training set is 0.921, which shows that the diagnosis prediction model is very effective (Figure 8C). Moreover, the AUC value in the external validation set is 0.806 and there is no difference between the two datasets (*p* = 0.443) (Figure 8C). In conclusion, the logistic regression model established based on the four mRNAs and the two lncRNAs could effectively distinguish whether the sample type is ARVC or not, and *XIST*, *LINC00173*, *FBN1*, *COL1A1*, *COL5A1*, and *BGN* were potential targets for ARVC study.

## 4. Discussion

Although there has been a more comprehensive understanding of the pathology, genomics, and diagnostic methods of ARVC in the past two decades, the pathogenesis of ARVC is still unclear [23]. In the research of ARVC, genetic research is particularly prominent. At least 25 gene mutations have been found to be related to ARVC, such as DSP, PKP2, and DSG2 which are encoding related proteins that compose desmosomes, and DES which encodes the desmin intermediate filament protein [7,24]. Due to the large number of mutated genes, the genetic heterogeneity between individuals is significant. Diagnosis by relying solely on mutated genes has high specificity but low sensitivity. Therefore, such a method is not yet reliable in clinical practice. From the perspective of transcriptomics, next-generation sequencing has expanded the disease beyond the classic desmosomal genes. In addition to comparing ARVC with non-failing donor hearts to identify differentially expressed genes, the researchers also compared ARVC with other cardiomyopathy such as DCM, so as to discover the unique transcriptomic characteristics of ARVC. In order to improve the sensitivity of diagnosis, we did not use the transcriptome data of DCM patients for analysis, but compared the mRNA, lncRNA, and miRNA of ARVC and non-failing donor heart to explore new diagnostic biomarkers. The researchers compared the ACM samples of different pathogenic variants, and there are differences between these groups [25]. However, due to the insufficient number of bulk RNA-seq samples and limited genetic information that we currently have, we are currently unable to perform accurately subgroup analysis, but fortunately from the existing gene mutation data, samples of these data sets are not single gene mutation, they are all mixed, and have certain comparability. Through GO enrichment analysis, we found that these differentially expressed mRNAs are mainly enriched in terms such as extracellular matrix organization, collagen fibril organization, response to transforming growth factor beta, and SMAD protein signal transduction. Myocardial fibrofatty replacement is one of the main characteristics of ARVC. Fibrosis is a common feature of most cardiomyopathies, leading to systolic and diastolic dysfunction and an increased risk of ventricular arrhythmias [26]. Camilla Schinner et al. using gene set enrichment analyses revealed an upregulation of genes associated with TGF-β signaling in both 9-week-old DSG2 mutant mice as well as patients with ACM [27]. Notably, TGFβ3, one of the hub genes identified by us, which belong to the term “response to transforming growth factor beta”, is a non-desmosomal gene recognized in ARVC and its mutation is associated with ARVC pathogenesis [28]. Smad2/3 plays a signal transduction role in the downstream of TGF-β. TGF-β induces the expression of α-smooth muscle actin in cardiac fibroblasts, promotes the transformation of fibroblasts into myofibroblasts, and stimulates myofibroblasts to secrete extracellular matrix such as collagen [29]. Based on the above evidence, we speculate that the TGF-β/SMAD signaling pathway plays an important role in the process of ARVC fibrosis. Targeted intervention for this pathway in the future may reduce the degree of fibrosis of ARVC. Meanwhile, in addition to the classic involvement of the right ventricle, arrhythmogenic cardiomyopathy can also affect the left ventricle, resulting in pathological manifestations of fibrous fat replacing myocardial cells. Therefore, anti-fibrotic therapy is beneficial for both. Further research is needed on the left ventricular ceRNA regulatory network in ACM [30].

Combining DElnc and DEmiR with DEM, we constructed an lncRNA-miRNA-mRNA regulatory network in this study, and finally a total of four mRNAs (FBN1, COL1A1, COL5A1, BGN) and two lncRNAs (XIST, LINC00173) were identified. Through logistic regression analysis, these genes showed high sensitivity and specificity for the diagnostic prediction of ARVC. It further shows the potential role of the ceRNA network in the diagnosis of ARVC. These genes are essential for cardiovascular disease but have not been studied in ARVC. Among these mRNAs and lncRNAs, FBN1 (fibrillin-1) mutations are more common in patients with aortic dissection in Marfan syndrome [31]. Fibrillin-1 is one of the important components of the extracellular matrix. When there are frameshift mutations or missense mutations of its coding gene, misfolding of fibrillin-1 will cause structural disorder of the extracellular matrix [32]. A multi-level transcriptomics (mRNA, lncRNA, miRNA) sequencing of left ventricular samples from patients with end-stage heart failure identified COL1A1 as a biomarker for the prognosis of heart failure, and it was also verified at the plasma level [33]. COL5A1, encoding type V collagen, although it does not account for a large proportion of fibrous scars formed after heart injury, plays an important role in regulating the size of fibrous scars [34]. Experiments in mice show that knocking out Col5a1 increases the scar area after myocardial infarction [34]. Biglycan (BGN) is a key member of the leucine-rich small proteoglycan family and an important part of the extracellular matrix. A previous work analyzed a variety of human solid tumor transcriptome data, in which BGN was identified as a potential diagnostic and prognostic biomarker [35]. XIST is related to X chromosome silencing [36]. Bettina Heidecker et al. showed that the expression of XIST is elevated in patients with new-onset heart failure [37]. LINC00173 promotes the proliferation and migration of vascular endothelial cells, which is related to the angiogenesis and tumorigenesis of lung squamous cell carcinoma [38]. Although we validated the above results at the RNA level, we still lack protein-level validation. In the future, we can verify the trend of mRNA changes at the protein level. The above genes play a crucial role in cardiovascular disease, demonstrating their importance. Secondly, these genes are mainly associated with extracellular matrix and fibrosis, further demonstrating the importance of the ARVC fibrosis mechanism.

In our work, although the miRNAs were not used to construct a diagnostic prediction model, they are key nodes connecting mRNAs and lncRNAs in the ceRNA network. In the network, hsa-miR-21-5p, hsa-miR-144-3p, hsa-miR-29b-3p, and hsa-miR-10b-5p are up-regulated miRNAs; hsa-miR-320a, hsa-miR-494-3p, hsa-let-7b-5p, hsa-miR-149-5p, hsa-miR-122-5p, and hsa-miR-182-5p are down-regulated miRNAs. Among these miRNAs, miR-21-5p is also found to up-regulate in ARVC right ventricular tissue in another data set [13]. Conversely, miRNA profiling in the pericardial fluid of ARVC and healthy controls identified miR-21-5p down-regulated [17]. We speculate that the possible reason is that miR-21 has cell expression specificity. In addition, miR-320a, miR-144-3p, and miR-494 are differentially expressed in plasma of ARVC patients [14,15]. Cardiac mesenchymal stromal cells were shown to be the main source of fat cells in ARVC [39]. Johannes Rainer et al. profiled coding and non-coding transcriptome in human cardiac stromal cells from ARVC patients and healthy controls, and they identified miR-29b-3p significantly up-regulated, which potential contributes to the pathogenesis and phenotype maintenance of ARVC [40]. miRNAs in both tissue and plasma are closely related to ARVC, further underlining the importance of the ceRNA network in ARVC. However, the functions of some miRNA that we identified have not been clarified in ARVC, and we found in our ceRNA network that there are some common regulatory genes between them such as *DDAH1*, *EPHA4*, *COL1A1,* and *ENC1*.

These previous studies supported the practical application of this lncRNA-miRNA-mRNA regulatory network for diagnosis of ARVC. However, there are still some limitations in our study. First, we have not adopted any miRNA in the network to construct the diagnostic prediction model. The main reason is that there is currently no sequencing data for both miRNA, lncRNA, and mRNA of matching samples from patients. Second, there is a lack of other experimental validation of the differentially expressed RNAs in human samples or animal models. Third, fibrosis is so important in ARVC that other signals are covered in kinds of bioinformatic screening methods, and the understanding of ARVC pathogenesis in this study may be limited. Further investigation will be needed to explore the ceRNA mechanism in ARVC.

## 5. Conclusions

In conclusion, we used ARVC transcriptome data to screen and construct an lncRNA-miRNA-mRNA ceRNA network through bioinformatics methods. *XIST*, *LINC00173*, *FBN1*, *COL1A1*, *COL5A1*, and *BGN* were identified as key molecules in the network, which have potential diagnostic value and may be therapeutic targets for ARVC. For these key molecules, more research is needed to further clarify their role and clinical value in the pathogenesis of ARVC.

## Figures and Tables

**Table 1:** Summary of the expression datasets involved in our study.

Study	RNA Types	Sample Types	Total Number	Control-RV	ARVC-RV	Others	Platform	GSE Number
Jia Li et al. (2020) [22]	mRNA	myocardial tissue	5	5	0	none	GPL16791	GSE107156
	mRNA	myocardial tissue	9	0	9	none		GSE107475
	lncRNA	myocardial tissue	5	5	0	none		GSE107156
	lncRNA	myocardial tissue	9	0	9	none		GSE107475
Gaertner A et al. (2012) [18]	mRNA	myocardial tissue	38	6	6	6 ARVC-LV, 7 DCM-LV, 7 DCM-RV, 6 NF-LV	GPL570	GSE29819
Maria Bueno Marinas et al. (2020) [16]	miRNA	myocardial tissue	18	6	12	none	NA	NA

## Data Availability

The datasets generated and analyzed during the current study are available in the GEO datasets repository: https://www.ncbi.nlm.nih.gov/geo/ (accessed on 1 November 2021); GSE107475, GSE107156, GSE29819.

## Supplementary Materials

The following supporting information can be downloaded at: https://www.mdpi.com/article/10.3390/jcdd11060168/s1. Figure S1: Data preprocessing of the expression profiles. Figure S2: Select the best beta value of soft-thresholding powers in WGCNA analysis. Table S1: Differentially expressed miRNA list. Table S2: RT-qPCR primer sequence.

## Abbreviations

Arrhythmogenic right ventricular cardiomyopathy (ARVC); long non-coding RNA (lncRNA); micro RNA (miRNA); competitive endogenous RNA (ceRNA); Gene Expression Omnibus (GEO); differentially expressed mRNA (DEM); differentially expressed lncRNA (DElnc); Gene ontology (GO); Kyoto Encyclopedia of Genes and Genomes (KEGG); Molecular Complex Detection (MCODE); Weighted Gene Co-expression Network Analysis (WGCNA).
