# Supplementary material for: Identification of Potential lncRNA-miRNA-mRNA Regulatory Network Contributing to Arrhythmogenic Right Ventricular Cardiomyopathy
Source: J Cardiovasc Dev Dis. 2024 May 30;11(6):168. doi: 10.3390/jcdd11060168 (PMC11204167; doi:10.3390/jcdd11060168)
Supplement: Supplementary file 1 [file jcdd-11-00168-s001.zip › Supplement.pdf]

SUPPLEMENTARY MATERIAL

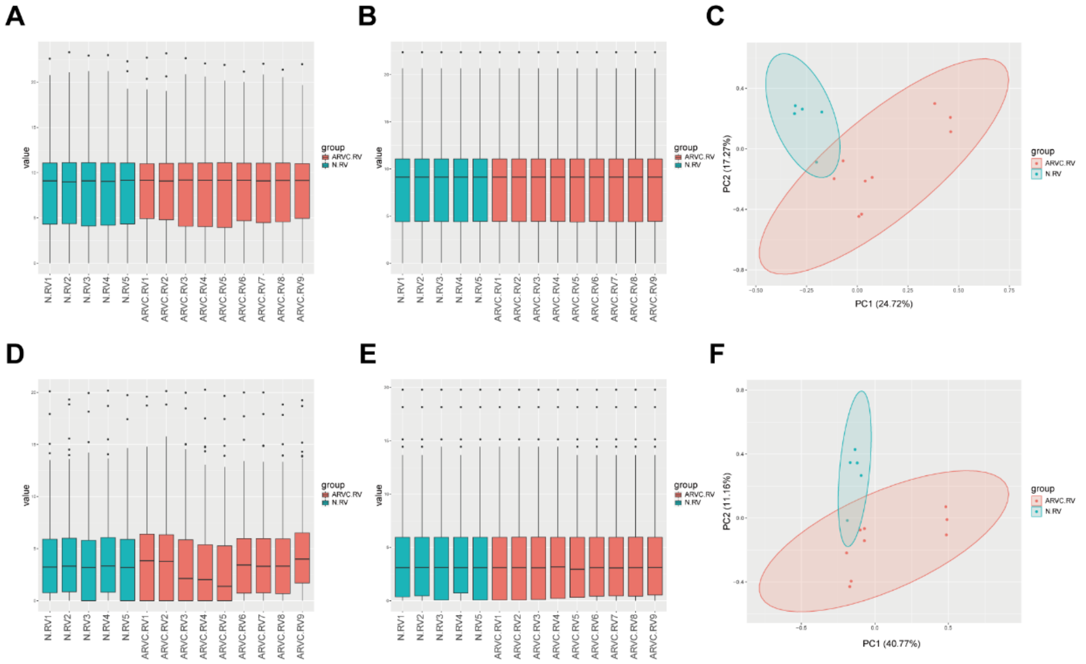

Figure S1. Data preprocessing of the expression profiles. A-C, Pre-normalization (**A**), post-normalization (**B**) and principal component analysis (PCA) (**C**) of mRNA expression profiles in GSE107475 and GSE107156 (Control n = 5, ARVC n = 9). **D-F,** Pre-normalization (**D**), post-normalization (**E**) and PCA (**F**) of lncRNA profiles in GSE107475 and GSE107156 (Control n = 5, PAH n = 9).

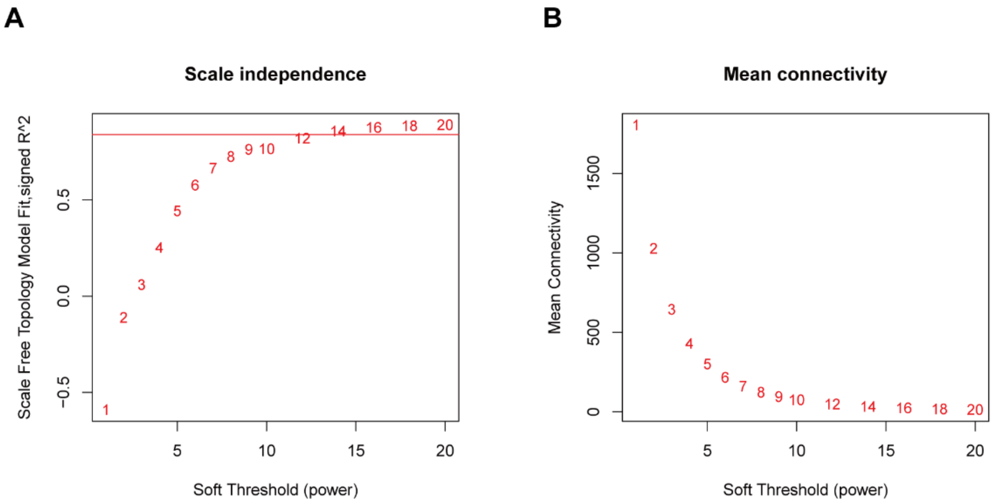

Figure S2. Select the best beta value of soft-thresholding powers. The scale-free topology model fit index (**A**) and the mean connectivity (**B**) for various soft-thresholding powers.

Table S1. Differentially expressed miRNA list.

Table S2. RT-qPCR primer sequence.
